# A Review of Compatibility Evaluation Methods and Improvement Measures Between Rubber Powder and Base Asphalt

**DOI:** 10.3390/ma19010139

**Published:** 2025-12-31

**Authors:** Dawei Wang, Peidong Du, Jiping Wang, Zhenqiang Han, Xiong Lan

**Affiliations:** 1 Guangxi Communications Investment Group Corporation Ltd., Nanning 530022, China; dawei1009@outlook.com; 2 School of Highway, Chang’an University, Xi’an 710064, China; jasonhan029@126.com; 3 Guangxi Rongwu Expressway Co., Ltd., Wuzhou 543300, China; huanglianhua139@126.com (J.W.);

**Keywords:** rubber powder, base asphalt, compatibility, activation treatment, storage stability

## Abstract

The increasing number of waste rubber tires has attracted the attention of more and more researchers. Rubber asphalt has better performance compared with original asphalt. However, the compatibility between rubber powder and asphalt is poor because of the difference in physical and chemical properties and the improvement of high-temperature performance of asphalt by rubber powder is very limited. The compatibility between rubber powder and original asphalt plays a key role in the storage stability and rheological performance of rubber asphalt. This paper provides a comprehensive overview of rubber asphalt, factors influencing compatibility, compatibility evaluation methods, and improvement approaches. Desired compatibility results from the fact that rubber powder is cross-linked with molecules of original asphalt and evenly distributed in the original asphalt, forming a homogeneous system. Starting from the preparation process of rubber asphalt, the best preparation process was summarized. Then the activity of rubber powder is improved by physical and chemical methods, or other additives are added to finally promote the formation of a cross-linking network structure between rubber powder and original asphalt. Rheological method is the most widely used method in compatibility evaluation, but it is gradually accepted by researchers to evaluate the compatibility by observing the molecular morphology of rubber asphalt.

## 1. Introduction

The disposal of waste rubber tires is considered to be one of the most significant challenges facing humanity in the 21st century. As a non-biodegradable polymer material, discarded rubber tires that are not properly disposed of not only occupy vast amounts of land resources but also pose multiple hazards to the ecological environment [1,2,3,4,5]. It is evident that tires stored in outdoor environments are susceptible to combustion due to the accumulation of heat, which results in the release of substantial quantities of toxic fumes and chemical compounds. Conversely, the presence of heavy metals and organic additives in tires can result in the leaching of these substances into soil and groundwater systems through rainfall, thereby causing long-term pollution [6,7,8,9]. According to statistics from the China Rubber Industry Association, China produces in excess of 1 billion rubber tires per annum [10]. The enormous production volume of rubber tires corresponds to an equally staggering amount of waste tires generated. How to achieve their resource utilization has become an urgent issue that needs to be addressed.

At present, the principal methods for the disposal of waste tires are landfill disposal, incineration, thermal energy utilization and physical–chemical recycling [11]. Despite the variety of disposal technologies for waste tires, the most common method remains landfill disposal. This not only wastes renewable resources but also creates an ever-growing stockpile of solid waste [12,13,14]. It is worth noting that as the modern tire industry continues to demand higher material performance, tire products are rapidly evolving toward greater strength, wear resistance, stability, and aging resistance. This, in turn, makes discarded tires more difficult to decompose in natural environments. Generally speaking, waste tires are primarily composed of vulcanized rubber and various reinforcing materials (such as steel wires and fibers). The cross-linked vulcanized rubber forms a network polymer composed mainly of C–C and C–S bonds, making it difficult to degrade. If carelessly piled up or discarded, it not only pollutes the environment but also represents a significant waste of valuable rubber resources [15,16].

Converting waste tires into rubber powder for asphalt modification is widely recognized as one of the most effective ways to achieve high-value resource utilization. The combined use of asphalt and rubber has a long history, with the earliest related patent applications dating back to 1843 [17,18]. Since then, a significant number of researchers have conducted related studies on rubber asphalt. Subsequently, numerous researchers have dedicated themselves to studying the preparation processes, performance characterization, and engineering applications of rubber-modified asphalt, enabling it to demonstrate dual advantages in both environmental friendliness and road performance. However, the poor compatibility between rubber powder and asphalt remains a key technical challenge that limits its widespread use. The presence of chemical bonds, such as C–S bonds, and the three-dimensional network structure of rubber powder hinder its uniform dispersion in asphalt, leading to phase separation. This, in turn, has an adverse effect on the storage stability and service performance of rubber-modified asphalt. Therefore, to improve the compatibility between rubber powder and asphalt and obtain rubberized asphalt with more stable performance, it is essential to enhance the compatibility between rubber powder and asphalt.

To systematically review research progress in this field, the advancement of rubber-modified asphalt technology is promoted, and the level of waste tire resource utilization is enhanced. This paper reviews methods for evaluating the compatibility between rubber powder and base asphalt, along with improvement measures. The paper consists of three main parts: (1) overview the fundamental properties of rubber materials and asphalt materials, along with their interaction mechanisms; (2) systematically summarize existing compatibility evaluation methods, including microstructural characterization, rheological property testing, and macro-performance metric assessment; and (3) a detailed review of commonly used compatibility improvement measures—such as rubber surface modification, asphalt component adjustment, and the addition of compatibility agents—is provided to offer guidance for relevant research and practical applications.

## 2. Overview of Rubber Materials and Asphalt Materials

Rubber asphalt is a composite material formed by blending base asphalt with rubber powder obtained from the grinding and processing of scrap tires. Its properties are determined by both the base asphalt and the rubber powder, while the interaction behavior and mechanisms between the rubber powder and asphalt significantly influence the performance of rubber asphalt. This section primarily introduces the characteristics of asphalt materials, the material properties of rubber powder, their interaction, and the interaction mechanism between rubber powder and asphalt.

### 2.1. Asphalt Material

**Figure 1:**
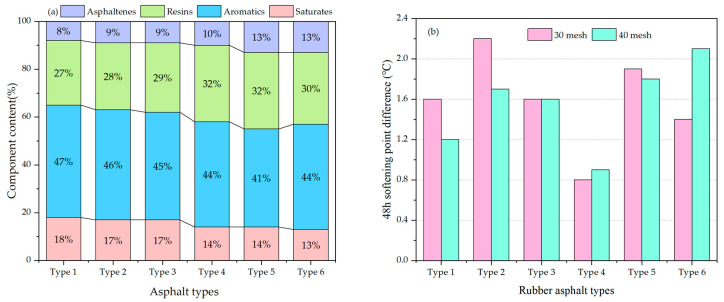
Asphalt composition and softening point difference: (**a**) Chemical composition test; (**b**) Softening point difference (based on [21]).

Asphalt is a viscous or solid mixture ranging in color from dark brown to black, composed of complex polymeric hydrocarbons and their nonmetallic (oxygen, sulfur, nitrogen) derivatives. Its composition is complex and its physical properties are variable, which significantly affects the compatibility of rubber asphalt. Researchers selected different types of asphalt materials and rubber powder for mixing and compounding, and determined the effect of different asphalt material compositions on compatibility based on physical and mechanical properties and viscosity tests [19,20]. Zhang et al. used six different types of asphalt to study the effect of asphalt composition on the compatibility of rubber powder and asphalt. Figure 1 shows the component analysis results of the six different types of asphalt [21].

The storage stability of rubber-modified asphalt is crucial to engineering quality, construction efficiency, long-term durability, and economic benefits throughout its entire lifecycle. Research has found that the chemical composition of base asphalt has a significant impact on the compatibility between rubber powder and asphalt. Among these components, resins and asphaltenes matter have a substantial negative impact on the storage stability of rubber asphalt, while saturates and aromatics have a relatively minor impact on the storage stability of rubber asphalt. Therefore, in order to improve the storage stability of rubber asphalt, when selecting asphalt, it is advisable to choose asphalt with lower resins and asphaltenes content and higher saturates and aromatics content. However, it should be noted that this rule does not apply to all types of asphalt. In addition to this, other factors such as the chemical composition and particle size of the rubber powder should also be considered when making a selection.

### 2.2. Rubber Material

**Figure 2:**
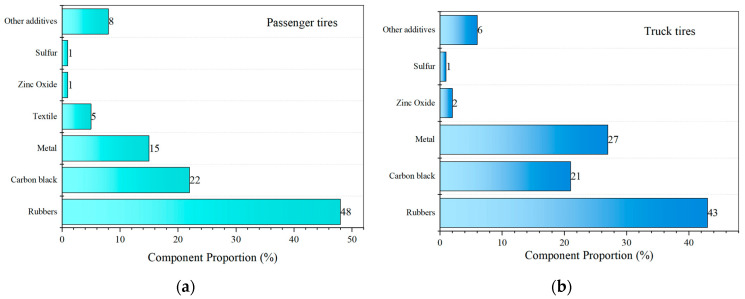
Typical components of vehicle tires: (**a**) Passenger tires; (**b**) Truck tires (based on [22]).

Rubber tires are principally composed of natural rubber, along with other reinforcing materials, and are produced through processes such as plasticization, mixing, and vulcanization. The primary components are illustrated in Figure 2.

Typically, waste tires undergo pretreatment processes such as magnetic separation, air classification, screening, and washing before being processed into rubber powder. These pretreatment processes remove fibers, steel wires, sand, moisture, and small-molecule organic compounds such as antioxidants and plasticizers added during tire manufacturing from waste tires.

The pretreated waste tires are then processed into rubber powder using a grinding method. Two primary methodologies are employed in the production of rubber powder from waste rubber tires: cryogenic grinding and ambient temperature grinding [23]. The cryogenic grinding method principally involves the freezing of waste rubber at sub-zero temperatures, followed by grinding at low temperatures. With the exception of the input of waste tires and the product packaging process, which involve contact with air, all other stages of the production process are conducted in a closed environment. Furthermore, the cryogenic grinding method serves to eliminate high-temperature odors and prevent secondary pollution. The heat exchange process between fine and coarse powders has the potential to optimize energy utilization and reduce energy consumption. The ambient temperature grinding method involves pre-processing waste rubber tires and then crushing them at ambient temperature. The crushed rubber blocks and powder are screened through a reciprocating screen at the bottom of the equipment. Once they meet the particle size requirements, they fall through the screen. Rubber blocks that do not meet the requirements are further crushed and ground. The ambient temperature grinding method has been shown to produce rubber powder particles of various sizes, which can be generated in different particle sizes according to requirements.

Hasson et al. investigated the surface morphology of rubber powder under different production processes [24]. Hasson’s study of SEM images of the microstructure of rubber powder produced by different grinding methods indicates: the production of rubber powder through crushing at ambient temperature results in a surface that is both rougher and possesses a larger specific surface area. This enhanced surface area facilitates the absorption of the light components present in asphalt to a greater extent. Consequently, rubber powder, produced through the process of crushing at ambient temperature, exhibits enhanced binding properties with asphalt, thereby demonstrating superior compatibility.

### 2.3. Mechanism of Interaction

Due to the complex composition of rubber powder and asphalt, and the complex physical and chemical reactions between their components, the interaction mechanism between rubber powder and asphalt during the mixing process is complex. Based on domestic and international research on rubber asphalt, the mechanism of interaction between rubber powder and asphalt can be summarized as follows [25,26,27].

1. Physical Blending Theory

The physical blending theory posits that when rubber powder and asphalt are mixed at high temperatures, the lighter components in the asphalt gradually permeate and diffuse into the network structure of the rubber powder particles under the combined effects of heat and agitation. This causes swelling, leading to an expansion in the volume of the rubber particles. The process primarily involves physical swelling, but under high temperatures and prolonged stirring, the swelling process intensifies, leading to partial dissolution and degradation. The dissolution process manifests as follows: under the influence of light components and heat, small-molecule substances within the rubber powder—such as uncrosslinked linear polymer chains, plasticizers, and anti-aging agents—separate from the rubber particles, diffuse, and dissolve into the surrounding asphalt matrix. The degradation manifests as follows: High temperatures and mechanical shear forces disrupt the main chains and cross-links of rubber molecules, causing chain-breaking degradation. The resulting rubber chains with reduced molecular weight also dissolve into the asphalt [25].

The swollen rubber powder forms a dispersion system with asphalt, with the rubber powder acting as the dispersed phase dispersed in the asphalt dispersion medium. The rubber powder particles gradually become larger, and the spacing between the particles decreases. The enlarged rubber powder intertwines with the asphalt to form a continuous three-dimensional network structure, thereby improving the performance of the rubber asphalt. The physical blending theory holds that rubber powder and asphalt are simply physically blended together, with weak bonding between the two. Consequently, rubber asphalt performs poorly and is susceptible to phenomena such as layer separation and segregation.

2. Chemical Blending Theory

The chemical blending theory holds that the interaction between rubber powder and asphalt is not merely physical blending, but also involves chemical reactions between the two [26]. The polar and nonpolar groups in asphalt undergo complex chemical reactions with the macromolecular chains of rubber powder, forming new chemical bonds and strengthening the bond between the two. In addition, during the preparation of rubber asphalt, the effects of heat and mechanical force cause the rubber powder molecules to break bonds, and the broken bonds undergo molecular chain rearrangement and reorganization with the macromolecular chains in the asphalt.

Generally speaking, as blending progresses, the interaction between rubber powder and asphalt gradually shifts from being primarily physical swelling to being primarily chemical bonding.

3. Network Filling Theory

The network filling theory suggests that, during the blending process, the light components in the asphalt first swell the rubber powder particles [27]. After swelling, the volume of rubber powder expands to 3–5 times its original volume, increasing the probability of contact between rubber powder particles. Rubber particles in contact with each other exhibit cross-linking and mutual interaction forces. The rubber powder particles cross-link with each other, thereby improving the physical and chemical properties of rubber asphalt.

It should be noted that this swelling process requires controlling the reaction temperature between 180 and 190 °C and maintaining the reaction time at approximately 45 min. The process must strictly control reaction temperature and duration, as prolonged exposure to high temperatures causes degradation and decomposition of rubber powder particles, resulting in reduced viscosity and deteriorated performance. Additionally, specific types of crosslinking agents (such as TOR) can be incorporated into the process to enhance high-temperature deformation resistance.

**Figure 3:**
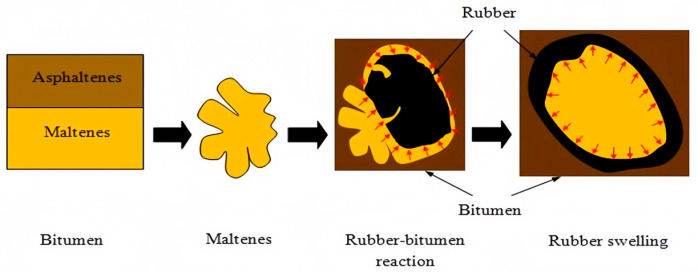
Rubber powder swelling model (based on [24]).

In summary, the above three theories may occur simultaneously during the mixing process of rubber powder and asphalt. At different reaction temperatures and reaction times, different mechanisms play a dominant role. Generally speaking, after rubber powder is added to asphalt, a swelling reaction occurs first, as shown in Figure 3. Rubber powder particles first absorb the light components in asphalt and swell. Asphaltenes are adsorbed onto the swollen particle surfaces. The swollen rubber powder particles form a semi-solid continuous network system through the asphaltene gel membrane on their surface. Ultimately, a structure is formed in which rubber powder particles are encapsulated in an asphaltene gel.

## 3. Evaluation Methods

In terms of thermodynamics, the compatibility between rubber powder and asphalt means that rubber powder particles can be evenly distributed in the asphalt matrix, forming a uniform system. However, in general, few materials can fully satisfy the thermodynamic compatibility conditions to form a homogeneous system. The differences in molecular weight and chemical structure between asphalt and rubber powder significantly affect the chemical and rheological properties of rubber asphalt. To qualitatively evaluate the compatibility between rubber powder and asphalt, researchers have proposed a variety of evaluation methods.

### 3.1. Storage Stability Testing

In accordance with the requirements of SH/T 0740-2003 [28], rubber asphalt should be filled into aluminum tubes with a diameter of 25 mm and a length of 140 mm. Then, position the aluminum tube containing the sample vertically in a constant-temperature oven maintained at 163 ± 5 °C for 48 h. Subsequently, place it in a refrigerator set at −20 ± 5 °C for 4 h to cool. After cooling, take rubber asphalt samples from the upper 1/3 and lower 1/3 parts, respectively. The compatibility of rubber asphalt is evaluated based on differences in the softening point, rheological properties, or microstructure of the upper and lower parts of the rubber asphalt [29]. In China, if the difference between the softening points of the upper and lower rubber asphalt is less than 2.5 °C, it is considered that the rubber powder is compatible with the asphalt. This method is currently considered one of the most authoritative methods for testing the storage stability of rubber asphalt and has been accepted by most scholars. Although this method is not highly accurate, it is easy to operate and provides intuitive results, which can provide experimental support and a theoretical basis for subsequent methods.

### 3.2. Rheological Methods

Compared with storage stability tests, rheological methods are more accurate in evaluating the compatibility of rubber powder and asphalt. The main rheological evaluation methods currently in use are the separation index method and the Cole-Cole plot method.

#### 3.2.1. Separation Index Method

The occurrence of phase separation causes significant changes in the resilient behavior of polymer-modified asphalt. Compared to storage stability tests, the separation index method can also predict the separation tendency between rubber powder and asphalt. Therefore, the separation index method has been widely used to evaluate the compatibility between rubber powder and asphalt.

Hussain U. B. proposed the ratio of the separation index of polymer-modified asphalt based on storage stability tests [30]. He suggested a scanning frequency of 0.15–15 Hz, maintaining a constant strain of 10% under high temperature conditions, and maintaining a strain level of 1.0% under ambient temperature conditions. The separation index R_S_ (Equation (1)) was calculated by measuring the complex shear modulus (G*) and phase angle (δ) of upper and lower polymer-modified asphalt samples. The lower the separation index (R_S_), the closer the properties of the upper and lower polymer-modified asphalt layers are, indicating better compatibility.

Wang et al. believe that the compatibility between polymers and asphalt can be evaluated using the irrecoverable flexural modulus J_nr_ measured by multiple creep stress recovery tests [31]. He believes that compared to G*/sinδ, the J_nr_ value can also reflect the strength and integrity of the polymer three-dimensional network structure in modified asphalt. A high-strength, continuous network structure can effectively resist external forces and elastically recover, thereby exhibiting a low J_nr_ value. The J_nr_ value not only provides a qualitative assessment of polymer-asphalt compatibility but also effectively characterizes the microstructure of rubber-modified asphalt after storage. Based on this, Equation (2) was proposed for evaluating the storage stability of polymer-modified bitumen at 64 °C. Furthermore, based on the research of Wang et al., Xu et al. further proposed an exponential calculation method for J_nr_ at 3.2 kPa (Equation (3)) [32]. The lower the SI value, the better the compatibility of rubber-modified asphalt.

Lang et al. employed the Maxwell model to fit the viscoelastic function of asphalt materials for the quantitative evaluation of rubber-asphalt compatibility. He tested the viscoelastic properties of polymer-modified asphalt at 50 °C through frequency scanning experiments, then substituted the results into the Maxwell model. Subsequently, he evaluated the compatibility between the polymer and asphalt based on the calculation results from Equation (4) [33,34].

Gonzalez used a temperature scan test, which tested the tangent of the loss angle (tanδ) at a constant frequency of 10 rad/s between 35 °C and 70 °C [35]. The experimental results were processed by fitting to obtain a temperature stability index (Equation (5)) to characterize the compatibility of polymer-modified asphalt. The higher the temperature stability index, the closer the ratio of elastic to viscous components in the polymer-enriched and asphalt-enriched zones, indicating good compatibility between the rubber powder and asphalt.

Table 1 summarizes the primary evaluation indicators and calculation methods of the separation index method.

#### 3.2.2. Cole-Cole Plot Method

**Figure 4:**
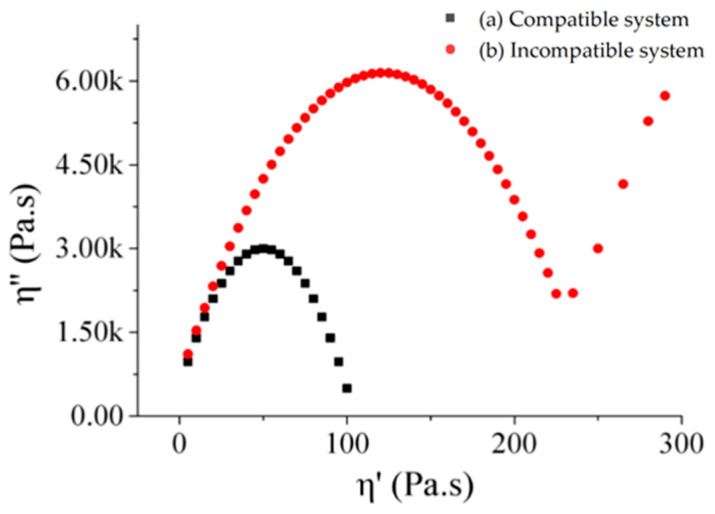
Cole-Cole plots in different systems [40].

The Cole-Cole plot is a curve composed of the real part (η′) and imaginary part (η″) of the dynamic complex viscosity measured by frequency sweep testing. Utracki’s research found that, compared to viscosity curves, Cole–Cole curves are more sensitive to phase separation and can provide relevant information about relaxation mechanisms [36]. Yvonne pointed out that Cole-Cole plots can be used to evaluate the compatibility between polymers and asphalt [37]. As shown in Figure 4, when the polymer-asphalt blend system is below the phase separation temperature, it exhibits a single arc curve (Figure 4—a). This curve indicates that only one relaxation mechanism exists in the mixed system, which also demonstrates the good compatibility between the polymer and asphalt [38]. However, when the phases separate, two peaks appear in the Cole-Cole plot (Figure 4—b). These two peaks represent two different substances, and as the temperature rises, the curves will no longer remain semicircular arcs. Therefore, the peaks of the curves in the Cole-Cole plot can be used to evaluate the compatibility between polymers and asphalt. At the same time, the potential tendency for polymers to separate from asphalt is characterized by whether the curve deviates from the semicircular arc [39].

### 3.3. Microstructure and Morphology

The degree of dispersion of polymers in asphalt is an important factor in characterizing the compatibility of polymer-modified asphalt. Compared with conventional macro tests, the microstructure and morphology of polymer modifiers and base asphalt can clearly and intuitively show the morphology of polymers in base asphalt. Consequently, this approach has become a widely accepted methodology for the assessment of compatibility between polymers and base asphalt.

#### 3.3.1. Solubility Parameter

Solubility parameters are physical constants that measure the miscibility of liquid materials. The dissolution and fusion process of polymer materials is essentially the process of interaction between solute molecules and solvent molecules. The free energy in the polymer dissolution process can be expressed by Equation (6):

(6) $$\Delta G_{M}=\Delta H_{M}-T\Delta S_{M}$$

where ΔG_M_ is the free energy of the mixing system; ΔH_M_ is the mixing enthalpy; T is the dissolution temperature of the system; and ΔS_M_ is the mixing entropy (ΔS_M_ > 0).

The solute will only dissolve in the solvent when ΔG_M_ < 0. Since the dissolution of solutes is an endothermic process, ΔH_M_ > 0. Therefore, the smaller the ΔH_M_ value, the more easily the two substances mix. ΔH_M_ can be calculated using Equation (7):

(7) $$\Delta H_{M}=\left|\frac{N_{a}V_{a}\cdot N_{b}V_{b}}{N_{a}V_{a}+N_{b}V_{b}}\right|\cdot \left|\sqrt{\frac{\Delta E_{a}}{V_{a}}}-\sqrt{\frac{\Delta E_{b}}{V_{b}}}\right|$$

where N_a_ and N_b_ are the relative molecular masses of polymers a and b; V_a_ and V_b_ are the molar volumes of polymers a and b; ΔE_a_/V_a_ and ΔE_b_/V_b_ are the cohesive energy densities of polymers a and b, J/cm^3^.

The solubility parameter δ of a substance can be expressed as:

(8) $$\delta =\sqrt{\frac{E}{V}}$$

ΔH_M_ is always greater than 0. In order to ensure that ΔHM is as small as possible, it is necessary to ensure that Δδ = |δ_a_ − δ_b_| is as small as possible. That is to say, it is necessary to ensure that δ_a_ and δ_b_ are as close as possible. It has been demonstrated by a considerable number of studies that there is a direct correlation between the solubility parameters of two substances and their compatibility. The closer the solubility parameters are, the greater the compatibility. Therefore, the solubility parameter can be used to evaluate the compatibility between polymers and asphalt.

#### 3.3.2. Microstructure

Scanning electron microscopy (SEM), fluorescence microscopy (FM), and atomic force microscopy (AFM) have been widely used to test the morphology of polymer-modified asphalt binders. These methods can clearly show the profile, size, and volume expansion of polymer modifiers in base asphalt [41]. Microanalysis suggests that rubber particles absorb light components from the asphalt binder and expand rapidly. If the rubber particles are uniformly distributed based on the rubberized asphalt binder and form a continuous phase structure, it means that the system is uniformly stable. It also indicates that the required compatibility has been achieved.

#### 3.3.3. Molecular Dynamics Simulation

Molecular dynamics is a numerical simulation method used in materials science. It has been widely used in the simulation of asphalt micro-component development. It is also used to simulate the modification mechanism of polymers on asphalt and the interaction between asphalt and aggregates. Guo et al. established models of cis-polybutadiene rubber (BR), styrene-butadiene rubber (SBR), and natural rubber (NR) using Materials Studio 8.0 software [42]. These models were used to calculate the solubility parameters and interaction energies of different types of rubber and asphalt at ambient temperature (298 K) and production temperature (453 K) to assess their compatibility. The results showed that all three rubbers were compatible with asphalt at production temperatures. The difference in solubility parameters between BR and asphalt is only 0.097 (J/cm^3^)^1/2^. The magnitude of the solubility parameter difference indicates that BR has the best compatibility with asphalt, followed by SBR and NR. Molecular dynamics simulation methods can intuitively and quantitatively evaluate the compatibility between polymers and asphalt, and have broad application prospects in the future.

In summary, this section reviews the current methods for evaluating the compatibility of rubber powder with base asphalt. It is evident that all of the aforementioned methods can be utilized for the purpose of evaluating the compatibility of diverse categories of rubber powder with base asphalt. Among them, the separation index method can not only evaluate the compatibility between rubber powder and base asphalt, but also predict their separation trend. This method can also effectively characterize the microstructure of rubber asphalt after storage while evaluating compatibility through specific test indicators. The peaks of the curves in the Cole-Cole plot are used to evaluate compatibility, and the degree of the curve deviation from the semicircular arc can also be used to characterize the potential tendency for separation between the polymer and asphalt. Therefore, rheological methods are currently the most widely used compatibility evaluation methods. The microstructure can more clearly and intuitively show the degree of dispersion and morphological characteristics of polymers in asphalt. In contemplating the future, the continuous advancement of computer technology will inevitably lead to computer simulation becoming a mainstream trend for assessing the compatibility between polymers and asphalt.

## 4. Measures to Improve Rubber-Asphalt Compatibility

Compatibility is one of the key indicators used to evaluate the performance of rubber asphalt, and it directly affects its performance. Generally speaking, the performance of rubber asphalt is positively correlated with the compatibility between rubber powder and asphalt. In the event of poor compatibility, the rubber powder will not disperse adequately in the asphalt, resulting in substantial disparities in the performance of the rubber asphalt. The inertness of the rubber powder surface and the significant difference in solubility parameters between rubber powder and asphalt are the direct causes of poor rubber-asphalt compatibility. Therefore, improving the compatibility of rubber asphalt is particularly important. This paper reviews the primary methods employed to enhance the compatibility between rubber and asphalt. There are four main types: the preparation process, physical methods, chemical methods and additive modification.

### 4.1. Preparation Process

#### 4.1.1. Rubber Powder Particle Size

In the production process of rubber powder, the size of the screen determines the final particle size of the rubber powder. Differences in rubber powder particle size lead to differences in rubber powder specific surface area. This further affects the reaction between rubber powder and asphalt. The California Department of Transportation conducted extensive testing using rubber powder particle size as the independent variable and rubber asphalt performance as the dependent variable. This research ultimately recommended a rubber powder particle size of 0.6 mm [43]. The research team at China University of Petroleum selected 48 h storage stability as the evaluation indicator to study the effect of rubber powder particle size on the compatibility of rubber and asphalt. The results showed that as the rubber powder particle size increased, the storage stability of rubber asphalt gradually deteriorated. Ultimately, a rubber powder particle size of 0.18–0.25 mm was recommended [44]. The evaluation methods employed by researchers are diverse, and the outcomes of these evaluations also exhibit variation. M. Capelo believes that the particle size of rubber powder should be around 0.212 mm [45]. Through experimental investigation, V. Chandran found that rubber asphalt has the best storage stability when the particle size of rubber powder is between 0.15 mm and 0.25 mm [46]. Liang et al. investigated the fluorescence microscopy images of rubber asphalt produced from rubber powders of different particle sizes. The white lines in his fluorescence microscope images represent rubber particles [44]. These rubber particles are not spherical, and their aspect ratio (length/diameter) increases with increasing particle size. When the rubber powder particle size is below 0.25 mm, the interaction between the rubber powder and asphalt is strong, indicating good compatibility. Due to variations in asphalt composition and differences in the sources of waste rubber tires, experimental results among different researchers have shown discrepancies. However, it is important to note that this discrepancy can be rendered negligible following the execution of specific data calculations and adjustments. Based on the results of numerous previous studies, this paper recommends that the particle size of rubber powder be between 0.15 and 0.25 mm in order to obtain rubber asphalt with good storage stability.

#### 4.1.2. Rubber Powder Content

Following the addition of rubber powder to asphalt, the initial phase is one of absorption of the light components in the asphalt, which subsequently undergoes a swelling reaction. The proportion of rubber powder directly determines its absorption capacity for light components in asphalt. It has been demonstrated that the degree of absorption of light components in asphalt is directly proportional to the rubber powder content [47]. This will also strengthen the interaction mechanism between rubber powder and asphalt, and theoretically improve the performance of rubber asphalt [48]. However, excessive rubber powder content will reduce the fluidity of asphalt and impair construction performance. Therefore, the rubber powder content should be controlled within an appropriate range to ensure optimal rubber asphalt performance. Different researchers use different rubber powders, asphalt, or evaluation parameters, resulting in different outcomes. The following are the optimal rubber powder content ratios obtained by some scholars through experiments: 18% [47], 10% [48], 15% [49], etc. Zhang et al. prepared rubber asphalt with different rubber powder content and tested indicators such as penetration at 25 °C, softening point, elongation at 5 °C, and elastic recovery. They ultimately concluded that the storage stability of rubber asphalt was best when the rubber powder content was between 15% and 25% [50]. Considering the comprehensive performance of rubber asphalt, as well as factors such as ease of construction and economic considerations, this paper ultimately concludes that the proportion of rubber powder should be controlled at around 20%.

#### 4.1.3. Preparation Technology

The preparation technology of rubber asphalt also has a certain impact on the performance of rubber asphalt. After the rubber powder is added to the asphalt, it gradually mixes with the asphalt under the action of shear force. The greater the shear rate, the greater the frequency of material exchange between the rubber powder and the asphalt. However, compared to shear rate, shear temperature has a more significant impact on the performance of rubber asphalt. As the magnitude of shear temperature is increased, a decrease in the viscosity of the asphalt is observed. Concurrently, an increase in the intensity of molecular movement is noted, thereby facilitating the diffusion of light components present within the asphalt into the rubber powder. However, a sustained rise in temperature will exacerbate the desulfurization of rubber powder and the aging of asphalt, thereby affecting the performance of rubber asphalt. Therefore, the selection of shear temperature also determines the performance of rubber asphalt. Currently, the processing temperature for rubber asphalt is primarily determined based on the fact that rubber powder easily disperses in asphalt but does not easily desulfurize [51,52]. Yu. M et al. used shear factors during the rubber asphalt preparation process as independent variables. Experiments were conducted by changing the shear temperature and shear rate, using penetration at 25 °C, softening point, elongation at 5 °C, and viscosity at 135 °C as evaluation indicators. The results showed that the shear rate had no significant effect on the performance of rubber asphalt, and the optimal shear temperature was determined to be 180–190 °C [53]. Sheng et al. tested and analyzed the Brookfield viscosity and rheological properties of rubber asphalt prepared at different temperatures. The results showed that the rubber asphalt performed best at a shear temperature of 170 °C [54]. Considering the performance characteristics of rubber asphalt and the complexity of its preparation process, this paper recommends that the shear temperature during rubber asphalt preparation be controlled at around 170 °C.

#### 4.1.4. Development Time

The preparation process of rubber powder modified asphalt can be roughly divided into three stages: swelling, dispersion, and development. The development time is critical to the production of modified asphalt. As the duration of the development time increases, the rubber powder particles undergo a process of swelling and degradation reactions, whilst the asphalt also undergoes a series of aging reactions. Therefore, different development times result in different dispersion states of rubber powder in rubber asphalt systems and different degrees of asphalt aging, which ultimately affect the performance of rubber asphalt [55]. Feng et al. investigated the effect of different developing times on the compatibility of rubber and asphalt [56]. He analyzed the microstructure of rubber-modified asphalt as a function of development time by capturing fluorescence microscope images of rubber-asphalt mixtures at 3 h, 6 h, 9 h, and 12 h development periods. Fluorescent microscopy images reveal rubber powder uniformly dispersed in asphalt in rod-like or strip-like structures. As the development time increases, the rubber powder particles continue to degrade in the asphalt, the particles gradually become smaller, and the dispersion state gradually becomes loose. This indicates that the compatibility between rubber powder and asphalt is improving. When the development time was longer than 9 h, there was little difference in the microscopic state of the rubber asphalt, and it was ultimately concluded that the optimal development time for rubber powder was 9 h.

### 4.2. Physical Methods

Rubber powder is formed by crushing and processing vulcanized rubber tires. Vulcanized rubber powder has low surface activity. When added to asphalt, it reacts poorly with small molecules in the asphalt. This reduces the interaction between rubber particles and asphalt, affecting the performance of rubber asphalt. Therefore, reducing the inertness of the rubber powder surface and reactivating the surface activity of rubber particles plays a decisive role in improving rubber-asphalt compatibility [57,58,59,60]. At present, the primary method for activating rubber powder is to subject it to a desulfurization process. This process serves to restore some of the properties of raw rubber to vulcanized rubber [61].

#### 4.2.1. Mechanical Desulfurization

Physical desulfurization of rubber powder refers to the process by which the C-S bonds within the rubber powder are reduced through physical treatment, thereby restoring certain properties of the raw rubber. Ultra-high-pressure water jet desulfurization is a method that uses the shear force generated by the bursting of bubbles during high-speed water flow to activate rubber particles. During the crushing process of rubber powder, the continuous breaking of bubbles erodes the surface of the rubber powder, making it rough and increasing its specific surface area [62]. After elemental analysis comparison, the internal S content of the desulfurized rubber powder was reduced by approximately 0.04%. Although this method produces rubber particles with smaller particle sizes, its desulfurization effect is quite limited [63]. Ultra-high-pressure water flow can break down rubber powder and remove sulfur to a certain extent, thereby improving the activity of the rubber powder, but the desulfurization effect is limited [64]. At the same time, considering economic efficiency, this method consumes a lot of energy and is therefore not recommended for use in actual production processes [65].

#### 4.2.2. Microwave Desulfurization

Mechanical desulfurization of rubber powder is only partial desulfurization, and the desulfurization effect is not ideal. In recent years, microwave desulfurization technology has attracted increasing attention from researchers. Microwave radiation interacts with material molecules through electromagnetic fields to generate energy, which is absorbed by the material and converted into heat. As a result, the temperature of the material rises rapidly, destroying the long molecular chains. Research shows that the temperature of the material can reach 260–350 °C during microwave treatment [66]. During microwave radiation, rubber powder can be treated by controlling the radiation time and frequency to break the C-S cross-linking bonds in the rubber powder while retaining the C-C bonds in the main chain, thereby achieving microwave desulfurization of the rubber powder [67]. Karima et al. investigated the effect of different microwave power levels on the desulfurization efficiency of rubber powder. He employed a household microwave oven equipped with a dual-emission system. Under microwave power conditions ranging from 350 to 900 W, the samples were subjected to electromagnetic microwave radiation for 15 s and 60 s, respectively, corresponding to microwave specific energy E. The test results showed that even at microwave radiation power levels of 900 W, the C-C bonds in the main chains of rubber powder molecules remain intact [68]. Presti used microwave-treated rubber powder as a substitute and tested the physical properties of the new rubber powder, finding that the physical properties were not affected by the treatment [69]. Movahed subjected rubber powder to microwave radiation at different temperatures and found that the desulfurization efficiency of rubber powder increased with rising temperatures [70]. Seghar et al. added pyrrolidine sulfate to the microwave desulfurization process of rubber powder and found that pyrrolidine sulfate has a high efficiency in converting microwaves, effectively promoting the desulfurization of rubber powder by microwaves [71]. Microwave desulfurization is highly effective and produces rubber powder with good performance, but it requires high energy consumption and emits large amounts of polluting gases during the process.

#### 4.2.3. Microbial Desulfurization

To avoid the drawbacks of microwave desulfurization, some researchers have used microorganisms that selectively reduce the crosslink density inside rubber powder by consuming the C-S bonds inside the rubber powder without damaging the C-C bonds inside the rubber powder. Li used thiobacillus ferrooxidans to selectively phagocytose the C-S bonds within the rubber powder. By comparing the solubility parameters of rubber powder before and after degradation, it was found that the solubility parameters of degraded rubber powder increased by 2.73%, and its physical properties also improved [72]. Li used thiobacillus activated rubber powder and found that after thiobacillus treatment, the oxygen content on the surface of the rubber powder increased by 30%, and the proportions of S-S bonds and C-C bonds decreased by 18.3% and 42.3%, respectively [73]. Yao et al. treated latex with a microorganism called *Alicyclobacillus* sp. After 10 days of cultivation, the S and O elements on the latex surface decreased by 62.5% and 34.9%, respectively, and the content of S-O bonds on the latex surface increased [74]. After treating vulcanized isoprene rubber and vulcanized styrene-butadiene rubber with Gordonia amicalisa microorganisms for 20 days, Hu et al. tested the crosslink density of the two vulcanized rubbers and found that the crosslink density of vulcanized isoprene rubber and vulcanized styrene-butadiene rubber decreased by 13.7% and 22.1%, respectively [75]. Cui used two types of microorganisms, *Sphingomonas* sp. and *Gordonia* sp., to desulfurize rubber powder. Compared with single-microorganism desulfurization, the desulfurization effect of the mixture was stronger than that of single desulfurization [76].

#### 4.2.4. Ultrasonic Desulfurization

Ultrasound is a sound wave with a frequency higher than 20,000 Hz that can produce high-frequency expansion and contraction vibrations in a medium, thereby causing acoustic cavitation. Therefore, under certain temperatures and pressures, high-frequency ultrasonic energy can be concentrated on molecular chains to produce a resonance reaction, converting ultrasonic energy into internal energy to rapidly destroy the three-dimensional network structure of rubber powder, thereby achieving desulfurization of rubber powder. Xu et al. used ultrasound to activate rubber powder and found that the crosslink density of the activated rubber powder was significantly reduced [77]. Compared with the above desulfurization methods, ultrasonic desulfurization of rubber powder is more efficient, does not produce any industrial wastewater or waste gas, and has a significant effect on the activation and modification of rubber powder. However, ultrasonic activation of rubber powder may cause excessive energy in localized areas of the rubber powder, resulting in the destruction of C-C bonds within the rubber powder.

#### 4.2.5. Low-Temperature Plasma Desulfurization

Low-temperature plasma technology is a rapidly developing surface modification technique for materials. This method enhances the compatibility with asphalt by improving the functional group characteristics of material surfaces through collisions with high-energy plasma—such as charged particles, excited particles, and photons generated by electrical discharges. Researchers used low-temperature plasma to activate rubber powder and compared the infrared spectra and scanning electron microscope images of the rubber powder before and after activation. The results indicate that low-temperature plasma activation treatment increases the number of hydroxyl groups on the rubber powder surface and renders the treated rubber powder surface rougher, thereby enhancing compatibility with asphalt [78].

### 4.3. Chemical Methods

#### 4.3.1. Surface Activation Modification

The activation modification of rubber powder mainly utilizes other reagents to improve the inertness of the rubber powder surface and enhance the reactivity between rubber powder and asphalt. Due to the strong corrosiveness of NaOH, many researchers use NaOH to pre-treat rubber powder. The surface of rubber powder treated with NaOH is rougher, and the storage stability of the rubber asphalt made from it is improved to a certain extent [36,51,79,80,81].

Silane coupling agents possess both inorganic and organic functional groups and are commonly used as polarity modification materials for particles to improve their interfacial properties [82,83,84]. Researchers used silane coupling agents to modify rubber powder and found that silane coupling agents can form a silane coupling film on the rubber surface. This film can react with double bonds or sulfides on the surface of rubber powder particles to form strong chemical bonds, thereby producing chemical adsorption. This aids in connecting the rubber powder with asphalt to form a stable, continuous system [85,86]. In addition, bio-oil, waste lubricant by-products, etc., can also be used to activate the surface of rubber powder, all of which can produce rubber asphalt with relatively good performance [87,88]. Surface-activated rubber powder modified with activators, the activation efficiency of the activators, and the degree of penetration of the activators into the rubber powder all play a critical role in the activation of the rubber.

#### 4.3.2. Polymer Coating Modification Method

**Figure 5:**
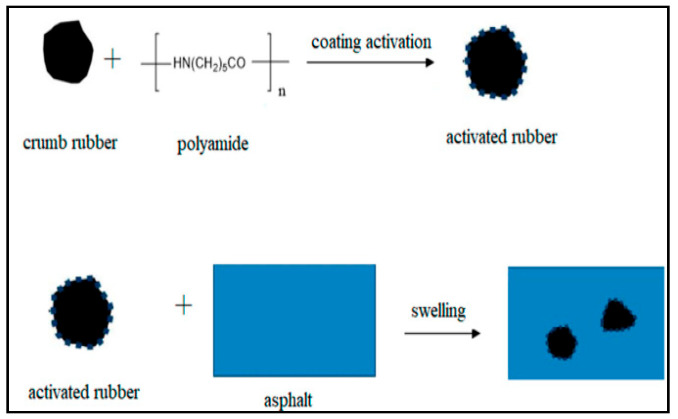
Schematic diagram of polyamide 6 modified rubber asphalt [90].

Polymer coating modification is a method of improving the activity of rubber powder by uniformly coating it with polymer using certain special methods. Yin et al. immersed microwave-treated rubber powder in epoxy soybean oil. Micro-performance testing revealed that epoxidized soybean oil can coat the surface of rubber powder, and the epoxy groups in epoxidized soybean oil can also adhere to the surface of rubber powder [89]. Yu et al. treated the surface of rubber powder with silane coupling agents and found through scanning electron microscopy and infrared spectroscopy analysis that a thin film of coupling agent had successfully coated the outer surface of the rubber powder [86]. Xie et al. evenly coated polyamide 6 on the surface of rubber powder to prepare rubber asphalt, and the performance of rubber asphalt was also significantly improved [90]. The modification mechanism of polyamide 6-coated activated modified rubber asphalt is shown in Figure 5.

#### 4.3.3. Core–Shell Modification Method

**Figure 6:**
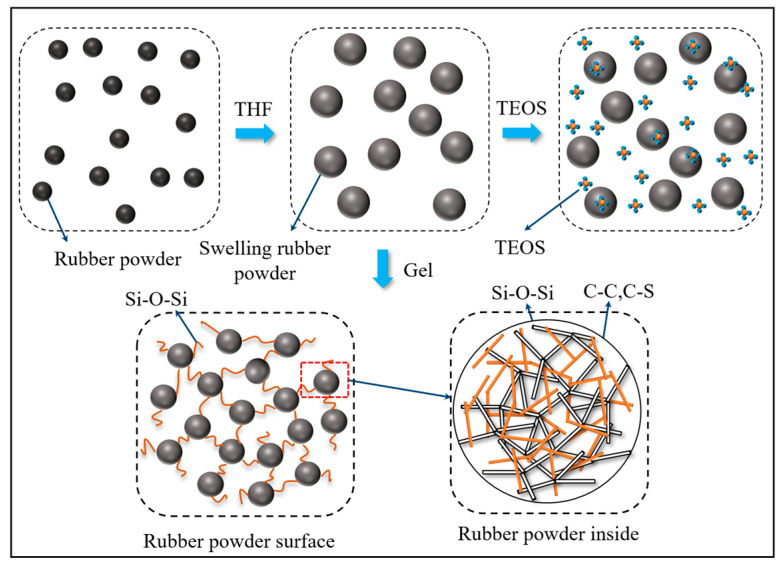
Mechanism diagram of TEOS modified rubber powder (based on [92]).

The core–shell modification method is a method of thoroughly modifying rubber powder from the inside out, divided into core modification and shell modification. Core modification mainly improves the cross-link density of rubber powder, with the aim of improving the uniformity of the rubber powder cross-link network, so that the rubber powder responds more uniformly to external forces. The main purpose of shell modification is to establish a reasonable transition layer between rubber powder and raw rubber, so that the cross-linking density of rubber powder gradually decreases from the inside to the outside, thereby preventing sulfur migration and increasing the activity of rubber powder [91]. Xu et al. used ethyl orthosilicate core–shell modification to prepare rubber asphalt and found that the storage stability of rubber asphalt was significantly improved [92,93]. Its mechanism of action is shown in Figure 6.

#### 4.3.4. Gas Modification Method

Gas modification is a method that uses active gases to activate the surface of rubber powder, thereby achieving the effect of modifying the surface of the rubber powder. Commonly used active gases include oxygen, bromine, chlorine, and carbon monoxide. After treatment, rubber powder particles often produce polar functional groups, thereby enhancing the surface activity of the rubber powder.

Combining the discussions in Section 4.2 and Section 4.3, Table 2 summarizes the specific measures for physical modification and chemical modification within rubber-asphalt compatibility improvement strategies, along with the advantages and disadvantages of each method.

### 4.4. Modification with Additives

In addition to optimizing the rubber asphalt preparation process and activating rubber powder, other additives can be added to enhance the bond between rubber powder and asphalt and improve the compatibility of rubber asphalt. The modification of rubber powder involves the condensation reaction between the chemical groups on the surface of the rubber powder and the monomer molecules. The process of introducing new chemical groups can increase the reactivity of the rubber powder surface, thereby increasing the reaction between the rubber powder and the polymer.

#### 4.4.1. Trans-Polyoctenamer Rubber (TOR) Additives

TOR is a high-molecular-weight polymer with a double bond structure. Its low viscosity at high temperatures promotes the blending of rubber powder and asphalt. At the same time, the double bond structure can undergo cross-linking reactions with sulfur on the surface of rubber powder and in asphalt, significantly improving the compatibility between rubber powder particles and asphalt. A large number of researchers have concluded through experiments and engineering practice that adding TOR as an additive to rubber asphalt mixtures can not only effectively improve the crack resistance of rubber asphalt, but also enhance its storage stability [67,94]. Researchers analyzed the effects of rubber powder content and TOR addition on the storage stability of rubber asphalt, indicating that TOR can effectively improve the swelling effect and stability of tire rubber powder and asphalt [95]. Liang et al. studied the performance evolution mechanism of rubber asphalt under multi-factor coupling effects and found that the cross-linked structure formed by uniformly dispersed TOR binders can significantly improve the stability and durability of rubber asphalt [96]. When TOR is added to rubber asphalt, the C=C double bond can connect small molecules in the asphalt with rubber powder molecules to form a network structure, thereby improving the compatibility between the two. However, TOR is a proprietary product that is relatively expensive and not suitable for widespread application.

#### 4.4.2. Eucommia Ulmoides Gum (EUG) Additives

EUG is added to rubber asphalt as a substitute for TOR to improve the compatibility of rubber asphalt. Deng et al. used a polybutadiene grafting method to graft maleic anhydride onto EUG, establishing a chemical bond between EUG and asphalt to increase the reactivity of rubber powder [97].

#### 4.4.3. Layered Double Hydroxide (LDHs) Additives

LDHs are supramolecular layered nanomaterials in which divalent metal cations at the top of the layers are replaced by trivalent cations, causing the layers to become positively charged. It adsorbs anions and water molecules between layers to compensate for the charge balance [98,99]. Liu et al. added LDHs as additives to rubber asphalt and observed the distribution of rubber powder on the surface of rubber asphalt using atomic force microscopy (AFM) [100]. The microstructure of rubber asphalt modified with LDHs is smoother and more uniform. This indicates that LDHs can effectively improve the agglomeration phenomenon of rubber powder. This is mainly because LDHs can form embedded or exfoliated structures with the molecular chains in rubber asphalt, increasing the cross-linking between rubber powder and asphalt. LDHs have a large specific surface area, high surface free energy, and irregular atomic arrangement at the interface. This characteristic enables LDHs to adsorb onto rubber powder during the mixing process with asphalt, thereby reducing the free energy of the system and inhibiting the agglomeration of rubber powder. This improves the bonding strength of the composite system interface and enhances the storage stability of rubber asphalt.

#### 4.4.4. Nano-Material Additives

Nanomaterials refer to solid materials with dimensions ranging from 1 to 100 nm, which typically exhibit unique physical and chemical properties not found in conventional materials. In recent years, nanomaterials have developed rapidly. Nanomaterials are characterized by their small size, high surface energy, large proportion of surface atoms, and large specific surface area. It also exhibits surface effects, small-size effects, quantum size effects, and macroscopic quantum tunneling effects [101,102,103,104]. Many researchers have attempted to combine nanomaterials with polymers to form composite materials in order to improve the performance of asphalt. Currently, the main nanomaterials used as modifiers for modified rubber asphalt are graphene, nano-organic montmorillonite (NOMMT), and nano-silica [105,106,107,108]. Due to the unique properties of nanomaterials, when added as modifiers to rubber asphalt, they not only impart unique properties to the rubber asphalt but also enhance the interaction mechanism between rubber powder and asphalt, thereby improving the performance of both rubber powder and asphalt.

## 5. Conclusions

This article provides an overview of the basic knowledge of rubber materials and base asphalt materials. This paper reviews the compatibility evaluation methods for rubber asphalt and provides a comprehensive overview of the methods for improving the compatibility between rubber powder and base asphalt. Based on these studies, the following conclusions were drawn:

1. The fundamental properties of raw materials are prerequisites for compatibility. The review indicates that base asphalts with lower resin and asphaltene content and higher saturate and aromatic content provide superior light components for permeating and swelling rubber powder, thereby enhancing compatibility. Meanwhile, rubber powder produced by ambient-temperature grinding exhibits superior performance in interaction with asphalt compared to low-temperature grinding rubber powder, owing to its more developed surface structure and lower degree of vulcanization network cross-linking.
2. Various evaluation methods each have their own characteristics, but the combination of rheological indicators and microstructure analysis is an effective approach to elucidating the compatibility mechanism. Storage stability testing is simple and straightforward, serving as an industry standard method; microscopic morphological analysis enables direct visual observation of the distribution state of two phases. Rheological methods can quantitatively characterize compatibility through multiple indicators, making them an intuitive and accurate approach. Future evaluation systems will trend toward the combined use of multiple methods and comprehensive judgment.
3. Process parameters exist within an optimal range rather than at discrete extremes. Research indicates that there is a significant interaction between the rubber powder content, particle size, shear temperature, and development time. To achieve optimal storage stability, the recommended process parameter range is as follows: rubber powder particle size 0.15–0.25 mm, compounding ratio 18–22%, shear temperature 170 ± 5 °C, and development time 8–10 h. Within this range, sufficient swelling degradation and interaction can be ensured while controlling energy consumption and aging risks.
4. Surface chemical activation represents a key approach to fundamentally improving compatibility, demonstrating significant potential in this regard. Surface activation of rubber powder (such as grafting and desulfurization) can break cross-linking bonds like C-S, thereby increasing surface energy and enhancing both chemical bonding and physical adsorption with asphalt. Although the effectiveness of various activation methods varies, the “hybrid process” combining chemical pretreatment of rubber powder with graft modification of additives in the asphalt matrix has been proven to synergistically enhance compatibility. This represents a rational and promising technical approach for improving the storage stability and long-term performance of rubber-modified asphalt.

## Figures and Tables

**Table 1:** Calculation method for separation index.

Equations	Evaluation Indicators	Parameter Definitions	References
Equation (1)	$R_{s}Ratio=\frac{G^{*}/\sin\delta_{top}}{G^{*}/\sin\delta_{bot}}$	G* is the complex shear modulus, δ is the phase angle	[30]
Equation (2)	$SI_{1}=\frac{{\left(J_{nr}\right)}_{top}-{\left(J_{nr}\right)}_{bot}}{J_{nr_{ave}}}$	Jnr is irrecoverable flexural modulus	[31]
Equation (3)	$SI_{2}=\frac{{\left(J_{nr}\right)}_{\max}-{\left(J_{nr}\right)}_{ave}}{J_{nr_{ave}}}$	Jnr is irrecoverable flexural modulus	[32]
Equation (4)	$I_{s}=\frac{\eta_{top}-\eta_{bot}}{\eta_{original}}$	η is the viscosity calculated by the Maxwell model	[33,34]
Equation (5)	$S_{i-T}=\sum_{T_{a}}^{T_{f}}\left[\left|1-\frac{\left(\frac{\tan\delta_{top}-\tan\delta_{bot}}{\tan\delta_{top}}\right)}{N}\right|\right]$	tan δ is the tangent of the loss angle, and N is the number of tests	[35]

**Table 2:** Comparison of rubber powder activation methods.

Modification Methods	Specific Measures	Advantages	Disadvantages
Physical methods	Mechanical desulfurization	Easy to operate, low instrument requirements	Low desulfurization efficiency
	Microwave desulfurization	High desulfurization efficiency	Generates exhaust gases and consumes high energy
	Microbial desulfurization	No wastewater or exhaust gas generated, low energy consumption	Longer activation time
	Ultrasonic desulfurization	High efficiency, no exhaust gas or wastewater generated	High energy consumption may damage macromolecules
	Low-temperature plasma desulfurization	Effectively activate rubber powder	High requirements for instruments
Chemical Methods	Surface activation modification	Effectively improves the surface activity of rubber powder	Complex procedures and expensive medicines
	Polymer coating modification		
	Core–shell modification		
	Gas modification		

## Data Availability

No new data were created or analyzed in this study. Data sharing is not applicable.
